# Individual Effects of Alkali Element and Wire Structure on Metal Transfer Process in Argon Metal-Cored Arc Welding

**DOI:** 10.3390/ma16083053

**Published:** 2023-04-12

**Authors:** Hanh Van Bui, Ngoc Quang Trinh, Shinichi Tashiro, Tetsuo Suga, Tomonori Kakizaki, Kei Yamazaki, Ackadech Lersvanichkool, Anthony B. Murphy, Manabu Tanaka

**Affiliations:** 1 School of Mechanical Engineering, Hanoi University of Science and Technology, Hanoi 100-000, Vietnam; hanh.buivan@hust.edu.vn; 2 Joining and Welding Research Institute, Osaka University, Osaka 567-0047, Japan; suga@jwri.osaka-u.ac.jp (T.S.); tanaka@jwri.osaka-u.ac.jp (M.T.); 3 Kobe Steel, Ltd., Fujisawa 251-8551, Japan; kakizaki.tomonori@kobelco.com (T.K.); yamazaki.kei@kobelco.com (K.Y.); 4 Thai Kobelco Welding Co., Ltd., Muang 10280, Thailand; ackadech.lers@kobelco.com; 5 CSIRO Manufacturing, Lindfield, NSW 2070, Australia; tony.murphy@csiro.au

**Keywords:** gas metal arc welding, metal-cored wire, metal transfer, alkali element, current path, metal vapor

## Abstract

This study aimed to clarify the effect of wire structure and alkaline elements in wire composition on metal transfer behavior in metal-cored arc welding (MCAW). A comparison of metal transfer in pure argon gas was carried out using a solid wire (wire 1), a metal-cored wire without an alkaline element (wire 2), and another metal-cored wire with 0.084 mass% of sodium (wire 3). The experiments were conducted under 280 and 320 A welding currents, observed by high-speed imaging techniques equipped with laser assistance and bandpass filters. At 280 A, wire 1 showed a streaming transfer mode, while the others showed a projected one. When the current was 320 A, the metal transfer of wire 2 changed to streaming, while wire 3 remained projected. As sodium has a lower ionization energy than iron, the mixing of sodium vapor into the iron plasma increases its electrical conductivity, raising the proportion of current flowing through metal vapor plasma. As a result, the current flows to the upper region of the molten metal on the wire tip, with the resulting electromagnetic force causing droplet detachment. Consequently, the metal transfer mode in wire 3 remained projected. Furthermore, weld bead formation is the best for wire 3.

## 1. Introduction

Since the gas metal arc welding (GMAW) process was invented and implemented, the transfer behavior of molten metal to the weld pool has been extensively investigated in many experimental and numerical studies due to its importance on the welding performance. For instance, Rhee and Kannatey-Asibu observed the metal transfer phenomenon for several transfer modes [1], and Liu and Siewert measured the transfer rate of molten metal to the weld pool in GMAW [2]. Hu and Tsai measured the arc characteristics and interactive coupling between the arc and molten metal using a unified comprehensive model [3]; furthermore, the melting of the electrode and droplet transfer regime was also predicted [4]. The literature concluded that the metal transfer was strongly influenced by the welding parameters, particularly welding current, shielding gas composition, and type of wire electrode [5]. The welding parameters naturally determine the strength of driving forces acting on the molten metal on the wire. Furthermore, auxiliary forces caused by laser irradiation [6], ultrasonic assistance [7], and wire movement [8] can be applied to improve droplet detachment.

The GMAW process can be carried out using a conventional solid or a tubular wire electrode. When the solid wire is used in pure argon shielding gas, the metal transfer mode is transformed depending on the welding current. In the free-flight transfer mode of the natural metal transfer class reported by Scotti et al. [9], the transfer mode can be classified as a globular, spray, or explosive transfer. The globular transfer was observed at a low welding current, in which the molten metal was transferred in large drops with a diameter greater than the wire diameter. The metal transfer was observed to change from globular to spray mode, with small droplets detaching at a high frequency when the welding current increases to a critical value [10]. In spray transfer mode, the metal transfer can be divided into projected, streaming, and rotating transfers according to increasing welding current [11]. The projected spray transfer is desirable due to a stable arc and metal transfer. A further high welding current can be applied when a welding process with a high deposition rate is required. However, the streaming and rotating transfer with an unstable arc and liquid metal transfer limits the welding current range, as reviewed by Paul et al. [12]. These studies imply that broadening the condition range to achieve a projected spray is advantageous; however, the mechanism to cause the transition of metal transfer mode is dependent on many factors and is not fully understood yet.

On the other hand, the welding process can also be carried out with a tubular wire, known as flux-cored arc welding (FCAW), to extend the application. The tubular wire consists of a metal sheath with flux powder inside. That configuration allows us to adjust the wire’s chemical composition or wire structure to improve weldability. For instance, Wang et al. evaluated the metal transfer of flux-cored wire through electrical arc signal, droplet diameter, and high-speed imaging measurement [13]. They reported that several different metal transfer modes could have coexisted simultaneously. Valensi et al. [14] found that the alkaline elements contribute to stabilizing the projected spray arc in an argon-CO_2_ gas mixture with 60%vol of CO_2_ at a 330 A welding current. Several fluorides, such as CaF_2_, KF, or K_2_SiF_6_, effectively control the hydrogen content contaminated in the weld bead; however, their presence causes an unstable arc with an undesired spatter, as reported in [15]. In addition, a wire with a high flux ratio was observed to reduce the amount of fume formation [16] and increase the metal transfer frequency [17]. The studies above implied that a tubular wire has a more complex metal transfer regime than a conventional solid wire.

Generally, the flux-cored wire can be classified into several groups, such as rutile, metal, basis gas-shielded electrode, and self-shielded electrode, depending on the flux formulation. The flux in the metal-cored wire type principally consists of iron powder to provide a high deposition rate. Starling and Modenesi [18] investigated the droplet transfer regime of three gas-shielded electrode types under 75% argon-25% CO_2_ and 100% CO_2_ shielding gas. It should be noted that the transfer behavior of metal-cored wire resembles that of solid wire. On the other hand, Trinh et al. [17] compared the weldability of a solid wire to three prototyped metal-cored wires in metal active gas (MAG) consisting of argon +20% CO_2_. They stated that the transfer frequency of the solid wire is much lower than that of the metal-cored wires, and the frequency increased with flux ratio and welding current. In addition, the effect of alkaline element proportion on metal transfer under MAG shielding gas was reported by Trinh et al. [19]. The result implied that an alkaline element could improve droplet detachment by forming an additional current path, bypassing the droplet to enhance the electromagnetic force to detach the droplet and reduce the arc pressure beneath the droplet. In the paper, the result was not compared with that for solid wire, so the effect of wire structure was not discussed. A previous study investigated the difference in the metal transfer between a solid and metal-cored wire in pure argon shielding gas [20]. It was reported that the metal-cored wire was not streaming transfer like the solid wire at a high welding current of 280 A. The difference can be explained by the fact that the presence of unmelted flux on the wire tip diminishes the formation of a liquid column necessary for a streaming transfer, which can be referred to as the effect of the wire structure. In this paper, a commercial metal-cored wire was used for MCAW, so the alkali element was not controlled as an experimental parameter.

As described above, the wire structure and the addition of an alkali element are considered to be two dominant factors to govern the metal transfer process in MCAW. However, the previous works [19,20] could not individually evaluate the effect of wire structure and alkaline elements. In this study, for the first time, the individual influences of wire structure and alkaline elements in metal-cored wire on the metal transfer behavior were evaluated. A solid wire, a metal-cored wire without an alkaline element, and a metal-cored wire with sodium as an alkaline element were investigated under a high welding current of 280 and 320 A, especially targeting the transition to streaming transfer in MCAW. The metal transfer behavior of the three wires was measured through a high-speed camera and spectroscopic observation. The comparison of the solid wire and the metal-cored wire without sodium can indicate the effect of wire structure; meanwhile, the comparison of the two metal-cored wires considers the impact of wire composition. Consequently, the mechanism of the metal transfer behavior was clarified.

## 2. Experimental Procedure

### 2.1. Materials and Welding Parameters

In the current work, welding experiments were produced on mild steel plates (SS400-JIS G 3101). The chemical compositions and mechanical properties of the base material are shown in Table 1 and Table 2, respectively. The dimensions of a workpiece are 300 mm × 50 mm × 9 mm. The main purpose of this paper is to clarify the metal transfer process, so bead-on-plate welding is carried out for simplicity as in [17,19,20]. To clarify the metal transfer behavior, three types of wire electrodes with a diameter of 1.2 mm were investigated, including a solid commercial wire and two prototyped metal-cored wires. The solid wire (wire 1) is the JIS Z3312 YGW11 wire, which corresponds to the A5.18 ER70S-G wire of the AWS classification. Meanwhile, the two metal-cored wires correspond to the A5.20 E70T-1C wire of AWS classification, including a wire without any alkaline elements (wire 2) and a wire with a flux containing 0.084 mass% of sodium (wire 3). According to production limitations, only the amount of sodium was controlled as an experimental parameter. Wire 3 is same as the wire 4 in [19], which has the largest amount of sodium of the prototyped wires. The details of the chemical compositions of the three wires are listed in Table 3.

**Figure 1:**
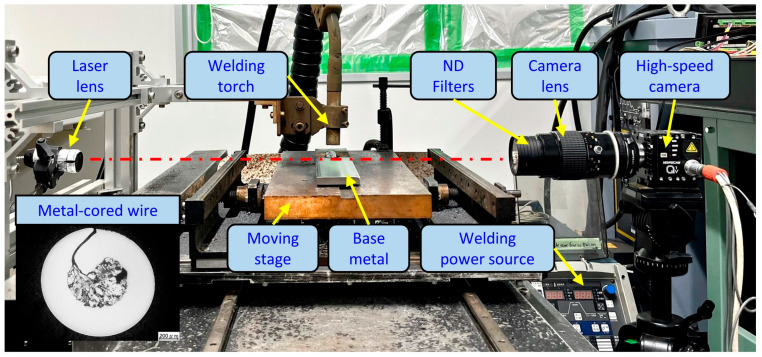
A photo of the experimental setup.

The welding process was conducted in direct current electrode positive (DCEP) mode using a DP-350 welding power source (OTC Daihen) equipped with an appropriate welding torch and wire feeder system. The metal transfer of three wires was investigated under two welding current levels at 280 A and 320 A. In this paper, we focused on a current range around the transition from globule (or project) transfer to streaming transfer for MCAW. The welding current was maintained among wires by varying the wire feeding speed for each wire. The welding voltage was adjusted to maintain a constant arc length of approximately 5.2 mm, for observing the metal transfer process clearly. During the welding, the welding torch was fixed, and the base metal was moved by an actuator. The details of the welding conditions are shown in Table 4. An experimental photo setup is depicted in Figure 1.

### 2.2. Visualization Procedure

In this study, a shadowgraph method was applied to observe the metal transfer behavior. The technique uses a radiation light source to illuminate an object from the opposite side of a camera, which allows the camera to obtain the shadow as the geometry of the object. The observation system consisted of a high-speed camera (Memrecam Q1v, Nac Image Technology, Tokyo, Japan), a camera lens (AF Micro-Nikkor, Nikon, Tokyo, Japan), and a laser system (Cavilux HF, Cavitar, Tampere, Finland). The laser system releases a laser from the laser lens to the camera lens, as shown in Figure 1. The camera was set to focus on the arc area, which obtains a clear image with a size of 640 × 480 pixels. The aperture value was f/4. Five neutral (ND) filters were utilized to reduce the intensive arc radiation. The observation conditions are summarized in Table 5.

In addition, a spectral observation technique was applied to investigate the arc properties in more detail. The camera was equipped with a bandpass filter, which allows light with a narrow range of wavelengths to go through. Two bandpass filters, appropriate to observe spectra of iron plasma for three wires and sodium plasma for wire 3, were applied, termed Fe I filter and Na I filter, respectively. The central wavelength of the two filters is presented in Table 2. The two filters had a full width at half maximum (FWHM) of 10.0 nm. In this experiment, the number of ND filters is three ND8, and the aperture was set at f/22.

## 3. Results and Discussions

### 3.1. Observation Results

**Figure 2:**
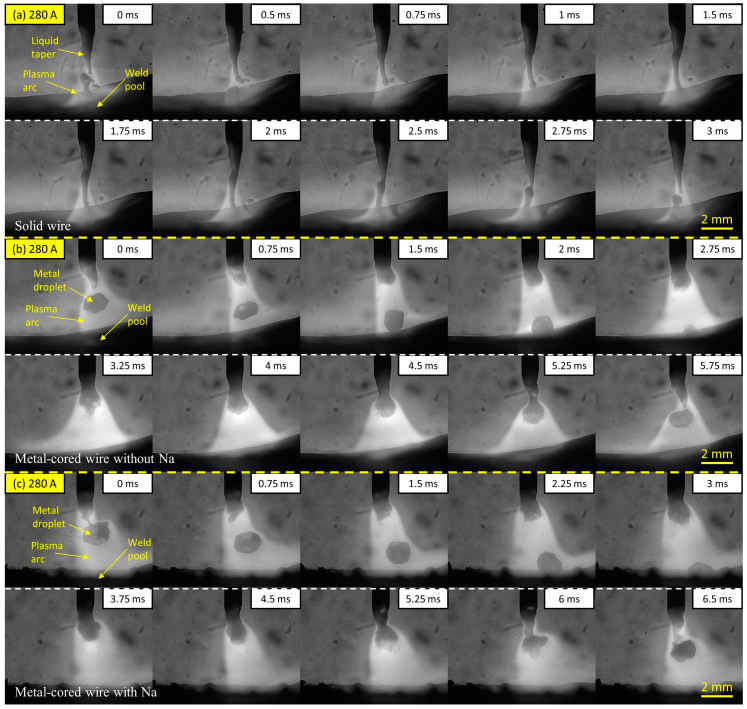
Metal transfer in wire 1 (**a**), wire 2 (**b**), and wire 3 (**c**) at 280 A welding current.

The observation results of the metal transfer of the three investigated wires at a welding current of 280 A are shown in Figure 2 as time-sequential images. The molten metal transportation and arc light concentration were captured simultaneously, which supports an evaluation of the arc attachment behavior on the wire tip during welding. The figure shows one cycle of the molten metal movement from the wire electrode to the weld pool. In Figure 2a, the metal transfer of the solid wire (wire 1) shows a streaming transfer in which a long liquid column in a pencil shape forms at the tip of the wire. The molten droplet was separated at the end of the column to contact the surface of the weld pool. At this welding current, the metal transfer of wire 1 in pure argon gas was reported in previous studies to be a streaming transfer mode [20,21]. The duration of one transfer in this condition is short at around 3 ms. However, due to the molten metal coming into direct contact with the molten pool unevenly, short-circuiting can occur between the end of the liquid column and the top of the molten metal droplet, as depicted at a frame of 0 ms. Consequently, the short-circuiting phenomenon resulted in a spatter, as observed in the period from frame 0.5 to 2 ms.

Figure 2b,c shows the metal transfer of the metal-cored wire without sodium (wire 2) and the metal-cored wire with sodium (wire 3), respectively. The droplet was transferred in a projected spray transfer mode in both circumstances. It can be observed that the droplet diameter was similar to the wire diameter. The times for a cycle transfer for wires 2 and 3 are 5.75 and 6.5 ms, corresponding to droplet transfer frequencies of approximately 174 and 154 Hz, respectively. For these two wires, in the duration of the droplet growth, it can be observed that the arc was attached at the neck position between the droplet and wire tip to cover the entire droplet. It can be considered that when the arc attachment is moved overhead of the droplet, a large part of the current is conducted directly from the neck of the wire to the argon plasma, avoiding the lower part of the droplet, because the dense iron plasma under the droplet has a relatively low electrical conductivity compared to that of argon plasma [22], so the electromagnetic force will act on the neck position effectively to enhance the droplet detachment. Meanwhile, the arc pressure in welding with pure argon gas was considered to be low. Thus, the droplet in metal-cored wires was transferred smoothly in projected mode. The reason for the metal transfer of metal-cored wire not to be streaming transfer was explained by Trinh et al. [20], in which two commercial solid and metal-cored wires were compared in pure argon shielding gas. It should be noted that during the arcing time, the unmelted flux column of metal-cored wire prevented the liquid column formation necessary for streaming transfer mode, particularly at 280 A welding current.

**Figure 3:**
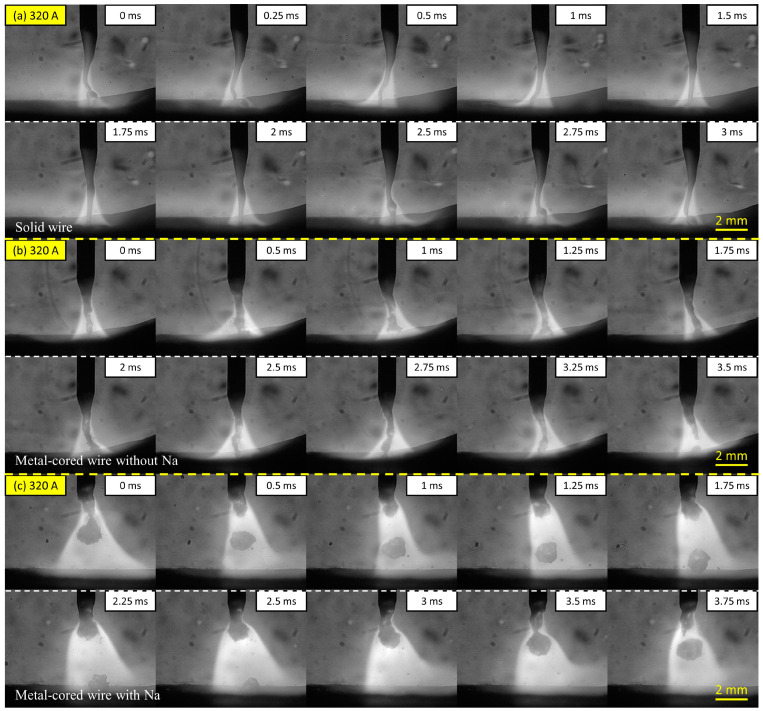
Metal transfer in wire 1 (**a**), wire 2 (**b**), and wire 3 (**c**) at 320 A welding current.

The metal transfer regime of the three wires at a high welding current of 320 A was compared in Figure 3. In Figure 3a, wire 1 shows a streaming transfer mode in the same manner as the transfer behavior in Figure 2a. At this welding current, the tip of the liquid column was extended to reach the surface of the base metal. The liquid column was thought to be related to the influence of electromagnetic force acting on the wire. To facilitate the droplet separation, the current following via molten metal on the wire tip must be sufficiently strong.

On the other hand, when a large proportion of current flows through the gas plasma rather than the molten droplet, the strong downward momentum of electromagnetic force squeezes molten metal on the wire tip to form a long liquid column [22]. When the welding current increases to 320 A, Joule heating becomes higher than 280 A, which increases the temperature to reduce the surface tension of the molten metal. As a result, the length of the column in 320 A was larger than that in 280 A. The tip of the column becomes slightly waved under the effect of driving forces, as shown in frames 0 to 1.5 ms. It was reported that when the current increases continually to around 400 A, the liquid column becomes unstable and rotates around the wire axis due to the strong electromagnetic force acting on the molten metal [23].

Figure 3b shows the images of metal transfer in wire 2 without sodium in the flux. The metal transfer mode is in streaming transfer, which is different from the projected transfer mode of this wire in 280 A, as shown in Figure 2b. A long liquid column was combined with a melted solid wire sheath and flux inside the wire for the streaming transfer in this wire. On the other hand, the metal transfer in wire 3 with 0.084% of sodium, shown in Figure 3c, was maintained in a projected transfer similar to 280 A. The cycle time for a droplet transfer is around 3.75 ms, corresponding to a transfer frequency of approximately 267 Hz. A molten droplet was generated and transferred under the arc attachment position during the projected transfer for this wire.

**Figure 4:**
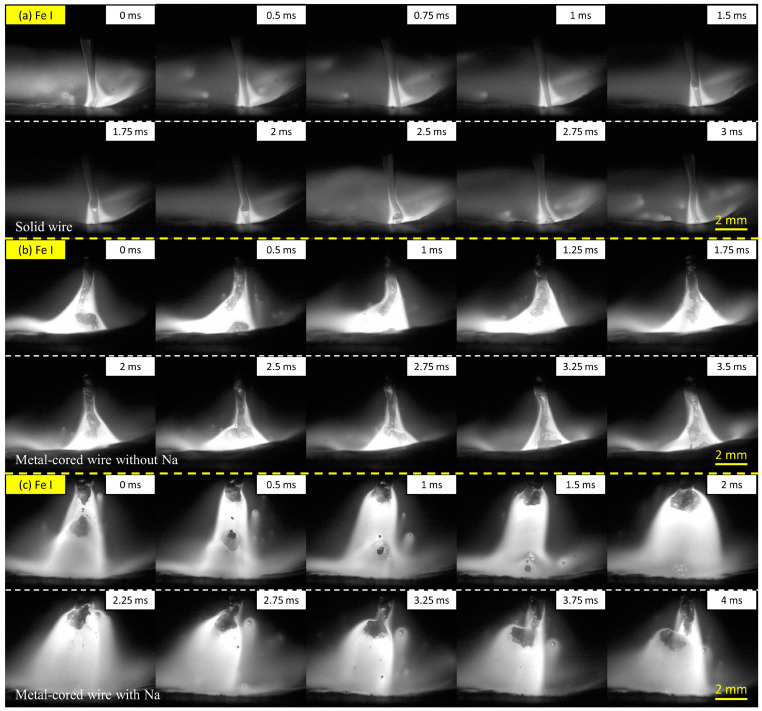
Metal transfer observation for wire 1 (**a**), wire 2 (**b**), and wire 3 (**c**) using the Fe I filter at 320 A of welding current.

This study also investigated distributions of iron plasma and sodium plasma at 320 A to clarify the difference in metal transfer mechanism among the wires. Figure 4 shows the iron plasma distribution obtained by the high-speed camera with the Fe I filter. In Figure 4a, the iron plasma in wire 1 was located around the tip of the liquid column, which generated iron vapor plasma in a conical shape from a position higher than the detachment point to the surface of the weld pool. The result is consistent with a previous study by Trinh et al. [20], in which the iron and argon plasma in the streaming transfer was observed to separate to form a dual structure in the arc plasma. Thus, the argon plasma in this circumstance was expected to cover iron plasma at the arc center. In Figure 4b, the iron plasma distribution of wire 2 is similar to that of wire 1 in streaming transfer.

In contrast, the iron plasma distribution of wire 3 in Figure 4c shows a different behavior. During the projected transfer, the iron plasma attached under the droplet from the beginning of droplet formation, as observed from frame 0.5 to 3.25 ms. Due to the smaller size of the molten droplet, the entire droplet was enveloped by iron plasma.

**Figure 5:**
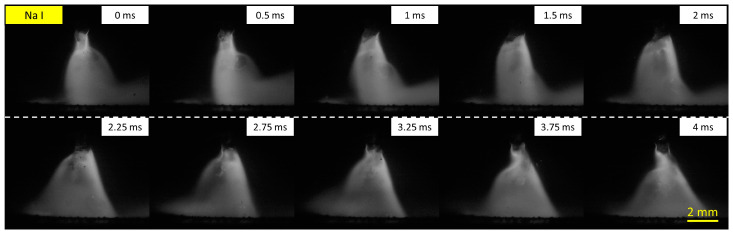
Metal transfer observation using the Na I filter for wire 3 at 320 A welding current.

Figure 5 shows time-sequential photos of metal transfer observation using the Na I filter for wire 3 at 320 A of welding current. It can be observed that the sodium plasma was distributed from the wire tip to the surface of the weld pool. In a previous study conducted by Trinh et al. [19], it was explained that very fine sodium particles were distributed in the wire flux before melting and injected into the arc intermittently to form sodium plasma after melting. At the droplet detachment moment, the sodium plasma under the wire tip was observed to be confined to a radially narrow region, as shown in frames 0 and 0.5 ms. During the period from 1 to 3.25 ms, sodium plasma was broadened from the wire tip and covered the droplet.

**Figure 6:**
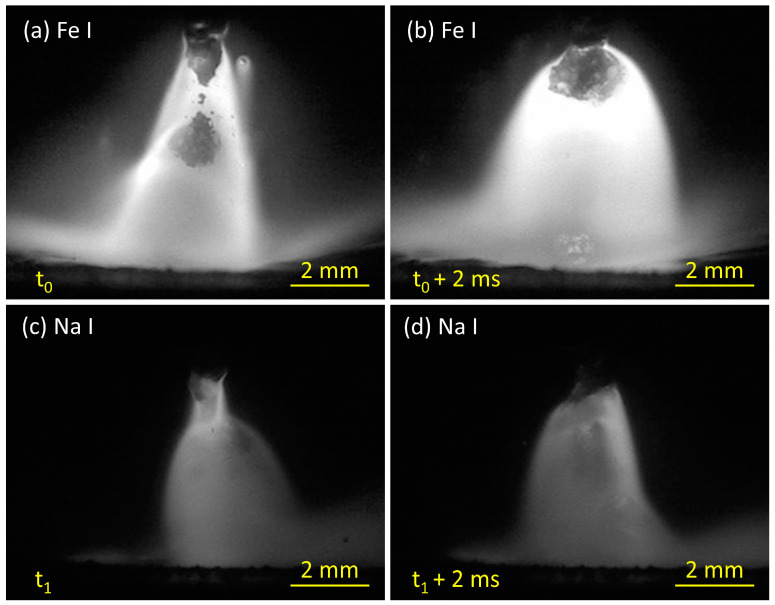
The typical images of iron vapor plasma (**a**,**b**) and sodium vapor plasma distribution (**c**,**d**) of wire 3 at 320 A of welding current.

Figure 6 compares the iron and sodium plasma distribution of wire 3 at 320 A. Figure 6a shows the iron plasma when a droplet was completely detached from the wire tip, defined as time t_0_, and Figure 6b shows the iron plasma at 2 ms after that moment. At the time t_0_, the iron plasma has an arc root around the tip of the unmelted flux. The iron plasma gradually expanded to envelop the entire droplet surface, as observed at t_0_ + 2 ms. In addition, Figure 6c,d show the sodium plasma distribution when another droplet detached from the wire tip, defined as time t_1_, and that at t_1_ + 2 ms, respectively. The sodium plasma was concentrated at the tip of the melted flux, as shown in Figure 6c. At the time t_1_, the sodium vapor evaporated from the melted flux was observed to be distributed radially narrower than the iron vapor. The sodium vapor broadened at the wire tip when the droplet size increased at the moment t_1_ + 2 ms, as shown in Figure 6d.

### 3.2. Metal Transfer Mechanism

**Figure 7:**
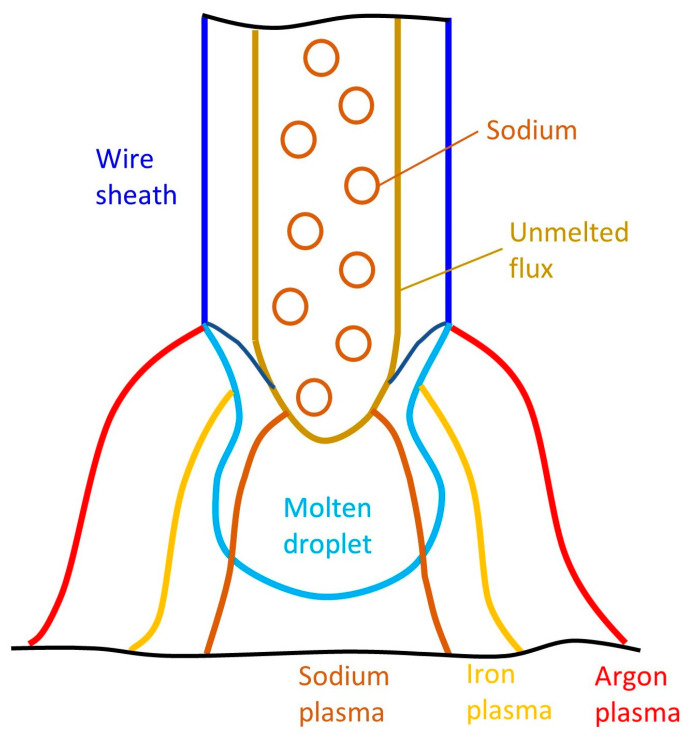
Position of plasma distribution for wire 3.

Based on the experimental results, a schematic of the arc plasma distribution for wire 3 was suggested in Figure 7. Three plasma regions were considered, including sodium, iron, and argon plasma. In metal-cored wire 3, sodium is added only to the flux inside the wire, which is enveloped by the wire sheath. Even though sodium has a significantly lower boiling point than iron (1156 K compared to 3134 K), sodium could not evaporate at a higher position than the solid part of the wire sheath covering the flux around the wire tip because the solid wire sheath is thought to prevent the evaporation. In addition, the arc attachment was reported to move upward when the iron intensively evaporated because the arc temperature was lowered by a substantial radiation loss, causing a reduction in the electrical conductivity of iron plasma [22]. It should be noted that the highest position of arc attachment corresponds to the taper part of the wire sheath, where the wire surface begins to melt by the intensive heat flux from the high-temperature arc and the electron condensation [24,25]. The iron vapor is considered to start the evaporation from this taper part, unlike the sodium vapor.

Furthermore, with pure argon shielding gas, the argon plasma was observed to be located at the top of the molten metal, and the iron plasma was connected closely below the argon plasma for welding a commercial metal-cored wire [20]. The commercial metal-cored wire contains some alkaline elements, such as potassium and sodium, which may lead the arc phenomena similar to wire 3 in this study.

Trinh et al. [19] found that sodium inside the wire assisted a new current path from the wire tip to the base metal to bypass the molten droplet. In that experiment, sodium was thought to evaporate from a higher position than iron on the wire tip because the investigation was conducted at a low current of 220 A in argon +20% CO_2_ shielding gas. Under that welding condition, the arc tended to constrict due to the high specific heat of CO_2_. The iron plasma was especially concentrated underneath the bottom of the droplet, lower than the position where sodium evaporated. In this study, pure argon was used as a shielding gas. At a high current of 320 A, the droplet size in the projected transfer was small, which enabled the iron plasma to reach the overhead of the droplet to attach to a higher position than sodium plasma. The arc plasma can be separated into the outer argon plasma and inner metal vapor plasma, in which metal vapor consists of the iron vapor covering the sodium vapor. Therefore, the sodium, iron, and argon plasmas were distributed as discussed above.

**Figure 8:**
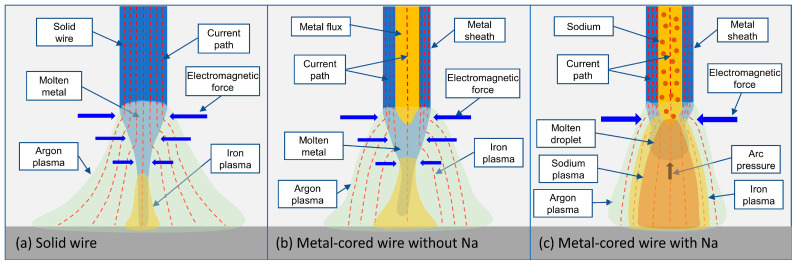
Mechanism of metal transfer (**a**–**c**) at a high welding current of 320 A.

Based on the evaluation in Figure 7, a mechanism comparing the metal transfer behavior of the three wires at 320 A welding current was suggested in Figure 8. In Figure 8a,b, the streaming transfer was observed in solid wire 1 and metal-cored wire 2 without sodium. In both circumstances, the argon plasma was located on the surface of the wire. It was reported that most of the current flowed in the argon plasma region owing to its high temperature. On the other hand, iron plasma has a lower temperature due to the strong radiation loss of iron vapor [21,22]. During the welding, the electromagnetic force acting on a molten droplet is significantly influenced by the current path that follows inside the droplet [26,27]. The direction of electromagnetic force in the wire is almost inward, because the axial component of the current is larger than the radial component. According to the separation into the argon and iron plasmas explained above, most of the electromagnetic force is considered to be applied above the iron plasma. When the current paths inside the droplet are insignificant, the electromagnetic force is not enough to separate the droplet from molten metal on the wire tip. As a result, the liquid metal at the wire tip was squeezed to form a long, tapered liquid column.

In Figure 8a, the taper extends to come into contact with the surface of the weld pool. In GMAW, the molten metal at the wire tip was heated by combining the Joule heating effect, thermal conduction from the high-temperature arc, and electron condensation at the surface of the wire. The combination of these heat sources strongly affects the wire melting phenomena and configuration of the wire tip [3,28]. As explained previously, the length of the liquid column in 320 A was larger than that in 280 A. In Figure 8b, the streaming transfer behavior of wire 2 was slightly different. In metal-cored wire, the low electrical conductivity of metal flux leads to most of the current flows inside the solid metal sheath; as a result, the flux was heated mainly from the molten metal sheath, flux, and arc by the thermal conduction. The unmelted flux was ineffective in liquid column formation, which increased the transition current to the streaming transfer in metal-cored wire 2.

**Figure 9:**
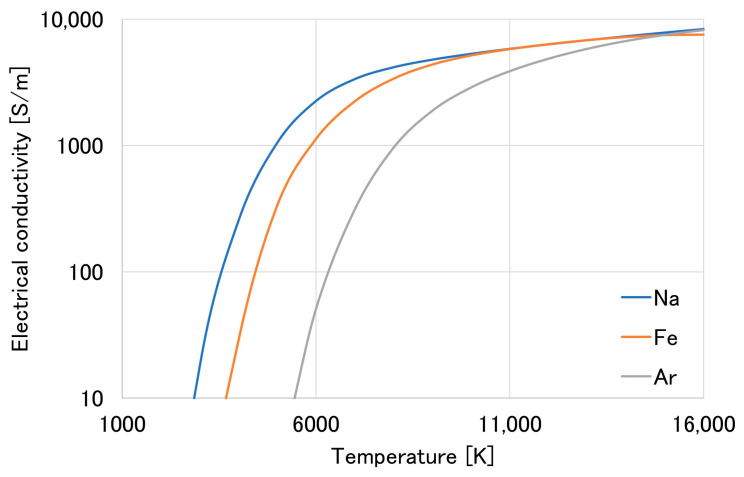
Electrical conductivity of sodium (Na), iron (Fe), and argon (Ar) plasma.

In Figure 8c, the metal transfer in the metal-cored wire shows a projected behavior. The droplet was transferred in a diameter less than the wire diameter. In addition, a comparison of the calculated electrical conductivities of the sodium, iron, and argon plasmas as a function of temperature is shown in Figure 9. The calculated value of iron and argon plasma was obtained, as discussed in [29,30]. At the same time, sodium vapor was applied in similar methods, with the momentum transfer for interactions between metal atoms and electrons [31]. The figure shows that sodium plasma has a higher electrical conductivity than iron, especially at a temperature range from 3000 to 7000 K. On the other hand, the electrical conductivity of argon plasma becomes significant at a high temperature above 10,000 K. Sodium and iron have ionization energies of 5.1 eV and 7.9 eV [32], respectively. The presence of sodium plasma in the arc will increase the current following through the metal vapor, thus increasing the current flowing out from the bottom of the droplet. As a result, the electromagnetic force applied on the molten metal and the arc pressure under the droplet increase. Consequently, the metal transfer mode of wire 3 was the projected transfer even in a high welding current.

### 3.3. Weld Bead Formation

**Figure 10:**
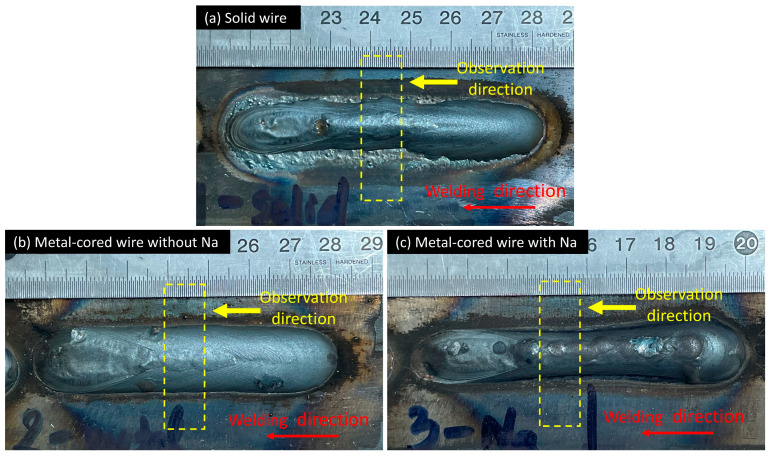
The photo of the weld bead appearance.

In this study, weld beads were collected after completing experiments to investigate the effect of metal transfer on the weld bead formation. Figure 10 shows the weld bead appearance of three wires at 320 A welding current. In Figure 10a, the weld bead of solid wire 1 shows the variation at the toe of the weld bead along the welding direction. It can be explained by the instability of the liquid column on the wire tip forming in the streaming transfer [33,34]. Before welding, the surface of the base material was ground to remove the oxide layer, which reduced the presence of oxygen in the arc area. In the GMAW process with solid wire in pure argon shielding gas, the arc and metal transfers were unstable due to the movement of cathode spots. This phenomenon can be limited by adding small amounts of oxygen to the shielding gas [35].

On the other hand, the weld bead of two metal-cored wires shows a smooth weld toe. In metal-cored wire, the flux contains oxides of iron and metal alloys, which has a role similar to oxygen in shielding gas to prevent cathode spot movement. In addition, a dashed rectangle in the photos implying a specimen position with observation directions for cross-section investigation is shown in Figure 10.

**Figure 11:**
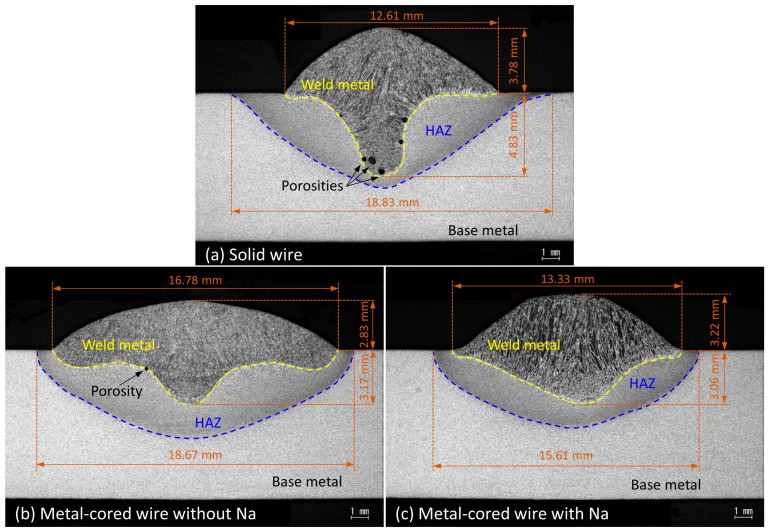
Weld bead cross-section.

Figure 11 shows the cross-section of three weld beads presented in Figure 10. The weld bead geometry of solid wire showed a finger shape, as observed in Figure 11a. In pure argon gas, the finger shape is a typical geometry of streaming transfer mode [36,37]. The cross-section shows many porosities located at the root penetration of the weld pool. It is considered that the long liquid column at the wire tip leads to molten metal transferred under the weld pool surface in streaming mode at 320 A, as observed in Figure 3a and Figure 4a. The submerged arc might cause the gasses to be trapped in the root of the weld bead after solidification. The cross-section of metal-cored wire 2 in Figure 11b shows a finger-like shape. The weld penetration in wire 2 was observed to be less than that in wire 1. It implies that the streaming transfer of wire 2 was less intensive than wire 1.

On the other hand, the cross-section of wire 3 in Figure 11c shows a shallow geometry. The deep penetration in this circumstance was similar to the bead of wire 2; however, the fusion line is smoother, and the heat-affected zone is narrower than that of wires 1 and 2 (15.61 mm compared to 18.83 and 18.67 mm, respectively). The narrower heat-affected zone implies that the heat input from the arc to base metal is smaller for wire 3, which is considered to be caused by a lowering of the arc voltage (the arc voltages for wires 1, 2, and 3 were 32.2, 33.8, and 31.2 V, respectively). As presented in Figure 9, the addition of an alkali element increases the electrical conductivity of the arc, thus the arc voltage might decrease. This decrease in heat input also leads to the lower wettability of the weld bead for wire 3 in Figure 10. The result indicates that welding with wire 3 has better weld stability than with the other.

The finding in this study implies that the sodium or alkaline elements can improve the arc stability by preventing the metal transfer not streaming at the high welding current. In addition, the impact of wire composition on the metal transfer is dominant other than the effect of the wire structure in GMAW with metal-cored wire.

## 4. Conclusions

This study investigated the effect of wire structure and wire composition of the metal-cored wire using high-speed camera observation. The comparison considered the metal transfer behavior for a solid wire, a metal-cored wire without alkaline elements, and another metal-cored wire with 0.084% mass of sodium in pure argon shielding gas under two welding currents of 280 and 320 A. The results lead to the following conclusions:

1. At 280 A, the solid wire showed a streaming transfer, and the other metal-cored wire showed a projected transfer mode. When the current increased to 320 A, the metal transfer of metal-cored wire without sodium changed to the streaming, while that of the metal-cored wire with sodium remained projected.
2. For metal-cored wire without sodium, the reason for the delayed transition to streaming transfer at 280 A corresponds to the wire structure. The unmelted flux with low electrical conductivity inside the wire limited the liquid column formation, which increased the transition current to streaming transfer.
3. In the metal-cored wire containing sodium, the iron plasma was observed to cover the sodium plasma. The presence of sodium plasma increases the current path through the metal vapor plasma region. As a result, the electromagnetic force acting on the neck of the droplet was more effective, and the arc pressure under the droplet increased. Consequently, the metal transfer is projected at 320 A welding current.
4. The results indicated that the effect on the welding stability of an alkaline element in the wire is larger than that of the wire structure for the metal-cored wire. Furthermore, the wire, including sodium, showed the best weld bead formation.

Our results provide strong evidence that the addition of sodium as an alkaline element to the wire is beneficial for metal-cored welding in argon at high currents.

## Figures and Tables

**Table 1:** Chemical compositions (mass%) of base material plates (JIS G 3101-2015).

Base Material	C	Mn	S	P
Mild steel SS400	–	–	≤0.050	≤0.050

**Table 2:** Mechanical properties of mild steel SS400 for 9 mm plates (JIS G 3101-2015).

Base Material	Yield Point (N/mm^2^)	Tensile Strength (N/mm^2^)	Elongation (%)	Bendability	
				Bending Angle	Inner Radius (mm)
Mild steel SS400	≥245	400–510	≥17	180°	13.5

**Table 3:** Chemical compositions (mass%) of wires.

Wire	Fe	C	Si	Mn	Cu	Al	Ti + Zr	Na
Wire 1	97.20	0.04	0.73	1.58	0.23	-	0.22	0
Wire 2	96.63	0.04	0.90	2.00	-	0.26	0.17	0
Wire 3	95.79	0.04	0.90	2.00	-	0.26	0.17	0.084

**Table 4:** Summary of welding conditions.

Parameters	Value/Unit
Welding current (output)	280 and 320 A
Welding voltage (output)	31.2–33.8 V
Shielding gas	Pure argon, 20 L/min
Contact tip to work distance	20 mm
Welding velocity	7 mm/s

**Table 5:** Summary of laser observation conditions.

Parameters	Value/Unit
Camera name	Nac, Memrecam Q1v
Frame rate	4000 fps
Shutter speed	20 µs
Aperture	f/4
Image	640 × 480 pixels
Laser wavelength	640 nm
The central wavelength of iron filter (Fe I filter)	540.0 nm
The central wavelength of sodium filter (Na I filter)	589.0 nm

## Data Availability

Data can be made available based on the requirements to verify this work.
